# Severe *Plasmodium falciparum* and *Plasmodium vivax* malaria among adults at Kassala Hospital, eastern Sudan

**DOI:** 10.1186/1475-2875-12-148

**Published:** 2013-05-01

**Authors:** Tajeldin M Abdallah, Mohamed T Abdeen, Ikhlas S Ahmed, Hamdan Z Hamdan, Mamoun Magzoub, Ishag Adam

**Affiliations:** 1 Faculty of Medicine, Kassala University, PO Box 496, Kassala, Sudan; 2 Faculty of Medicine, University of Khartoum, PO Box 102, Khartoum, Sudan; 3 Faculty of Medicine, Al-Neelain University, Khartoum, Sudan

**Keywords:** *Plasmodium falciparum*, *Plasmodium vivax*, Severe malaria, Sudan

## Abstract

**Background:**

There have been few published reports on severe *Plasmodium falciparum* and *Plasmodium vivax* malaria among adults in Africa.

**Methods:**

Clinical pattern/manifestations of severe *P. falciparum* and *P. vivax* (according to World Health Organization 2000 criteria) were described in adult patients admitted to Kassala Hospital, eastern Sudan.

**Results:**

A total of 139 adult patients (80 males, 57.6%) with a mean (SD) age of 37.2 (1.5) years presented with severe *P. falciparum* (113, 81.3%) or *P. vivax* (26, 18.7%) malaria. Manifestations among the 139 patients included hypotension (38, 27.3%), cerebral malaria (23, 16.5%), repeated convulsions (18, 13.0%), hypoglycaemia (15, 10.8%), hyperparasitaemia (14, 10.1%), jaundice (14, 10.1%), severe anaemia (10, 7.2%), bleeding (six, 4.3%), renal impairment (one, 0.7%) and more than one criteria (27, 19.4%). While the geometric mean of the parasite count was significantly higher in patients with severe *P. vivax* than with severe *P. falciparum* malaria (5,934.2 *vs* 13,906.6 asexual stage parasitaemia per μL, p = 0.013), the different disease manifestations were not significantly different between patients with *P. falciparum* or *P. vivax* malaria. Three patients (2.2%) died due to severe *P. falciparum* malaria. One had cerebral malaria, the second had renal impairment, jaundice and hypoglycaemia, and the third had repeated convulsions and hypotension.

**Conclusions:**

Severe malaria due to *P. falciparum* and *P. vivax* malaria is an existing entity among adults in eastern Sudan. Patients with severe *P. falciparum* and *P. vivax* develop similar disease manifestations.

## Background

Malaria is a big health problem and is endemic in an area where around two billion people live. *Plasmodium falciparum* causes approximately 600,000 deaths each year and the vast majority of the burden of malaria mortality is in sub-Saharan African countries [1]. The high mortality rate from *P. falciparum* is due to its ability to induce severe malaria, and in some cases, multiple organ dysfunction [2]. The presenting symptoms and mortality patterns of severe malaria vary widely according to the geographical setting and therefore transmission intensity. In areas with high, stable transmission in sub-Saharan Africa, severe anaemia in infants, which has a relatively good prognosis, is the main presentation, and severe malaria does not occur in adults with acquired immunity [2]. In areas with moderate transmission, cerebral malaria in young children is the most common presentation. In areas with low transmission, such as Southeast Asia, severe malaria occurs in all age groups including adults. Cerebral malaria, renal failure, severe jaundice, and pulmonary oedema are the main manifestations in the young adult population [2]. Yet the determinants of severe malaria and its pathophysiology are not completely understood. Therefore, epidemiological studies of severe malaria and its related deaths may provide additional understanding of its disease course, and, eventually, lead to improved case management. While many data have been published on severe malaria among children in Africa, there are few published studies regarding severe malaria among adult African patients [3-5]. Furthermore, these studies had a greater emphasis on *P. falciparum* malaria and there have been no published data on severe *Plasmodium vivax* infection among African adults. Until recently, *P. vivax* was considered a benign parasite compared with *P. falciparum*. The current study was conducted at Kassala Hospital in eastern Sudan to investigate the manifestations and epidemiology of severe *P. falciparum* and *P. vivax* malaria among adult patients, so as to add to the previous studies on severe malaria in Sudan [6-8]. Such data are of paramount importance for care-givers, health planners and researchers. The geographical area is characterized by unstable malaria transmission, and *P. falciparum* (95%) was the main species in the area. In contrast, *P. vivax* was considered rare and constitutes only 3% of the *Plasmodium* species in the area [9]. There is recent evidence that the rate of *P. vivax* malaria is increasing in this setting [10].

## Methods

**Figure 1:**
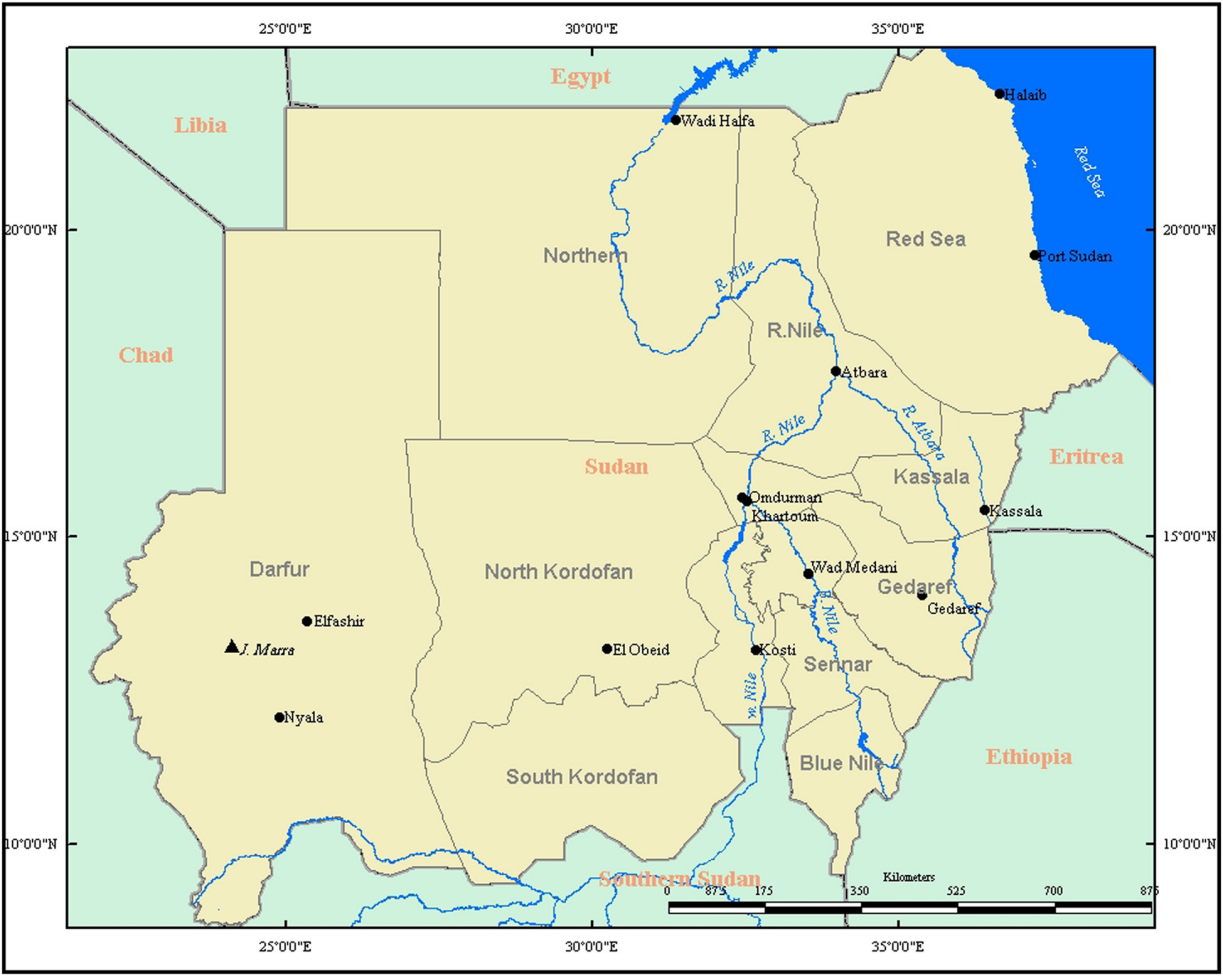
Map of Sudan.

A descriptive hospital-based study was conducted at Kassala Hospital, eastern Sudan, during two transmission seasons (between August and December, 2011 and 2012) to investigate the presentation/manifestation of severe malaria caused by *P. falciparum* or *P. vivax* (Figure 1).

All patients, or their relatives in the case of comatose patients, provided informed consent, and their socio-demographic and clinical data (temperature, weight and fever history) were collected using standard questionnaires. Patients with one or more manifestations of severe *P. falciparum* or *P. vivax* malaria according to World Health Organization (WHO) criteria were enrolled [2]. Pregnant women were not included. These criteria included cerebral malaria (unarousable coma and assessed by Glasgow coma scale), convulsions (more than two per 24 hours), hypotension (systolic blood pressure <70 mmHg with cold extremities), severe anaemia (haemoglobin <7 gm/dl), jaundice (detected clinically or bilirubin levels >3 mg/dl), hypoglycaemia (blood glucose <40 mg/dl) and hyperparasitaemia (parasite count >100,000 asexual stage parasitaemia/μl) as described by WHO guidelines, and the rest were considered as uncomplicated cases [2].

Thick and thin blood films were prepared from capillary blood, stained with Giemsa, and 100 oil immersion fields were examined. The parasite density was evaluated by counting the number of asexual parasites for every 200 leukocytes, assuming a leukocyte count of 8,000 leukocytes/μl. All slides were double checked in a blinded manner and only considered negative if no parasites were detected in 100 oil immersion fields. If gametocytes were seen, then the count was extended to 500 oil immersion fields. Haemoglobin concentrations were estimated using a HemoCue haemoglobinometer (HemoCue AB, Angelhom, Sweden). Blood glucose was measured at baseline before quinine infusion and two hours after quinine infusion if there was clinical suspicion of hypoglycaemia using the bedside device Accu-Chek™ Multiclix (Roche Diagnostics, Mannheim Germany). The Accu-Chek™ machine was calibrated weekly and a new box of test strips was opened each time.

Resuscitation and supportive management were given according to WHO guidelines [11] including correction of hypoglycaemia with 10% glucose, termination of convulsions with intravenous diazepam if they persisted for more than three minutes. Paracetamol was given every six hours until defervescence. Those with severe anaemia (haemoglobin <7 g/dl) and respiratory distress were transfused with blood screened for hepatitis and HIV. Vital signs were measured every 15 minutes for the first hour, then every two hours until 24 hours, and thereafter every six hours until patients were discharged from the hospital. Baseline investigations were performed for every patient upon admission and repeated when clinically indicated. These included measuring levels of haemoglobin, serum urea, serum creatinine, serum bilirubin and white blood cell counts.

Treatment during the first malaria season of 2011 consisted of quinine (quinine dihydrochloride, 20 mg salt/kg of loading dose, followed by 10 mg/kg three times a day over two to three hours, that was changed to oral quinine tablet when the patient could tolerate them). During the second season, intravenous artesunate was used with quinine in an open clinical trial. Artesunate was given as 2.4 mg/kg body weight at 0, 12, and 24 h, and then daily. Each 60mg vial of artesunic acid (Guilin, Pharmaceutical Factory, Guangxi, People’s Republic of China) was dissolved in 1mL of 5% sodium bicarbonate to form sodium artesunate and then mixed with 5mL of 5% dextrose. Details of the response to treatment will be described in a future report.

### Statistical analysis

Statistical analysis was performed using SPSS software (SPSS Inc., Chicago, IL, USA) version 16.0) and double checked before analysis. Data were checked for normality, and the Student *t*-test and analysis of variance were used for normally distributed data to compare between two or more than two groups, respectively. Proportions were compared by *χ*^*2*^-test. *P*-values <0.05 were considered significant.

### Ethical statement

The study received ethical approval from the Research Board at the Ministry of Health Kassala, Sudan.

## Results

**Figure 2:**
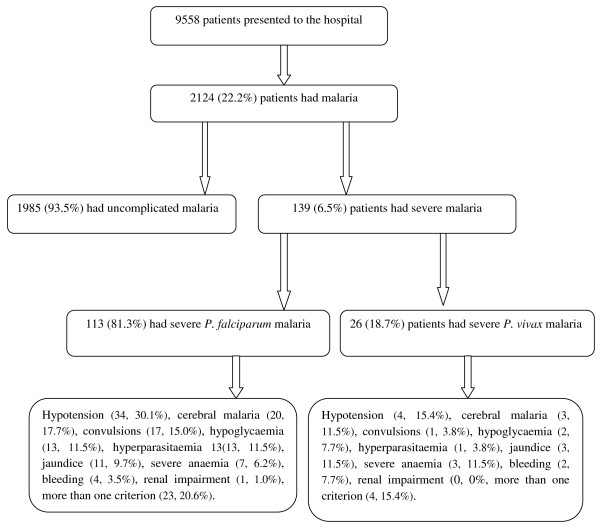
Clinical categorization of patients presenting with malaria-like symptoms.

Of the total 9,559 adult patients presented to the hospital during the study period, 2,124 (22.0%) patients had malaria. Among 2,124 patients with malaria, 1,985 patients had uncomplicated malaria, 139 patients (80 males, 57.6%) with a mean (SD) age of 37.2 (1.5) years presented with severe *P. falciparum* (113, 81.3%) or *P. vivax* (26, 18.7%) malaria (Figure 2). The ratio of severe *P. falciparum* to severe *P. vivax* malaria was 4.3 : 1.0. There were no cases of *P. falciparum* and *P. vivax* co-infection. There was no significant difference between the manifestations/presentations in patients enrolled in the 2011 season (55, 40%) and those in the 2012 season (84, 60.0%).

**Figure 3:**
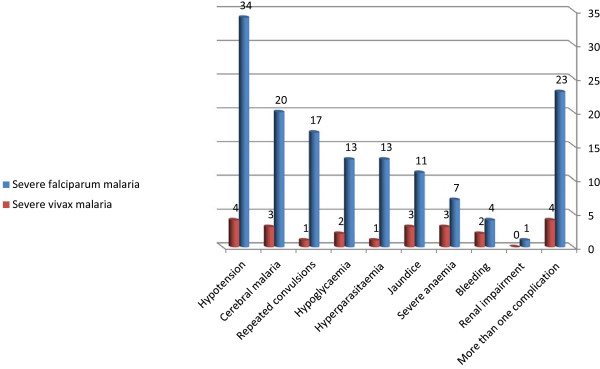
**Presentations of severe *Plasmodium falciparum *and *Plasmodium vivax *malaria.**

Among 139 patients with either *P. falciparum* or *P. vivax* infection, the frequency of symptoms were as follows: hypotension (38, 27.3%), cerebral malaria (23, 16.5%), repeated convulsions (18, 13.0%), hypoglycaemia (15, 10.8%), jaundice (14, 10.1%), hyperparasitaemia (14, 10.1%), severe anaemia (10, 7.2%), bleeding (six, 4.3%), renal impairment (one, 0.7%) and more than one criteria (27, 19.4%) (Figures 2 and 3). None of the patients had respiratory distress syndrome. The different manifestations of severe malaria were not significantly different between those infected with *P. falciparum* or *P. vivax* malaria (Table 1). The geometric mean of the parasite count was significantly higher in patients with severe *P. vivax* than in patients with severe *P. falciparum* malaria (5,934.2 *vs* 13,906.6; *P* = 0.013, Table 1).

**Table 1:** **The mean (SD) [range] of the clinical and biochemical data of patients with severe *Plasmodium falciparum *and *Plasmodium vivax *malaria**

**Variable**	***P. falciparum***	***P. vivax***	***P-value***
	**(Number = 113)**	**(Number = 26)**	
Age, years	38.0 (15.5)	34.2 (12.6)	0.260
	[18.0–78.0]	[17.0–70.0]	
Weight, kg	67.8 (13.4)	70.3 (11.0)	0.374
	[39.0–123.0]	[39.0–88.0]	
Duration of the illness, days	3.9 (3.6)	3.0 (1.6)	0.216
	[1.0–8.0]	[1.0–7.0]	
Axillary temperature, °C	38.4(0.8)	38.7 (1.4)	0.125
	[37.2–40.5]	[ 37.1–41.1]	
Systolic blood pressure, mm Hg	72.5 (23.4)	72.5 (13.8)	0.987
	[0–130.0]	[60.0–130.0]	
Diastolic blood pressure, mm Hg	47.0 (17.7)	50.0 (10.1)	0.414
	[0–100.0]	[30.0–70.0]	
Parasite count, rings/μl, Geometric	5,934.2	13,906.6	0.013
	[1600–380000]	[1500–115200]	
Haemoglobin, gm/dl	10.4 (2.6)	9.8(3.0)	0.302
	[3.6–14.2]	[3.8–13.9]	
White blood cells, cell/mm^3^	5,122.7 (1851)	5,507.7 (1,966)	0.346
	[1,900.0–11,400]	[1,800.0–9,000.0]	
Blood glucose, mg/dl	110.7 (44.3)	106.8 (28.3)	0.670
	[30.0–198.0]	[30.–170.0]	
Serum bilirubin, mg/dl	1.4 (1.0)	1.4 (1.2)	0.857
	[0.2–5.6]	[0.4–6.6]	
Serum creatinine, mg/dl	0.9 (0.5)	0.9(0.3)	0.738
	[0.1–4.6]	[0.5–2.0]	

Three patients presented with three manifestations of severe malaria as follows: patient 1: cerebral malaria, bleeding (epistaxis) and hypoglycaemia; patient 2: cerebral malaria with repeated convulsions and hypotension; and, patient 3: hypotension, severe anaemia and jaundice. Five patients developed severe anaemia and jaundice. There may be an overlap between the three main manifestations (hypotension, convulsion and cerebral malaria) of severe malaria. While there was no significant difference in levels of blood glucose, white blood cells and parasite counts between the patients presenting with hypotension, convulsion and cerebral malaria, their age (patients with cerebral malaria were older than those with convulsions, or hypotension) and haemoglobin were significantly higher in patients with cerebral malaria (Table 2).

**Table 2:** Comparison of clinical and biochemical characteristics of three main groups with severe malaria

**Variable**	**Convulsion**	**Cerebral malaria**	**Hypotension**	***P*-value**
	**(N = 18)**	**(N = 23)**	**(N = 38)**	
Age, years	39.5 (1.6)	43.0 (1.7)	36.4 (1.5)	<0.001
Haemoglobin, gm/dl	11.3 (3.1)	11.2 (2.1)	9.7 (2.2)	0.020
Blood glucose, mg/dl	106.5 (29.5)	123.2(33.1)	115.9 (54.0)	0.485
White blood cells, cell/mm3	5,650(1,804)	5,991(2,137.8)	5,229.0 (2,078)	0.361
Geometric mean of asexual stage parasitaemia per μL	4,339.7 (60,436)	7,986.7 (82,014)	5,831.9 (67,614)	0.986

Three patients (2.2%) died due to severe *P. falciparum* malaria. One had cerebral malaria, the second had renal impairment, jaundice and hypoglycaemia, and the third had repeated convulsions and hypotension.

## Discussion

The current study demonstrated a high ratio of *P. falciparum* to *P. vivax* malaria (4.3 : 1.0) in patients from eastern Sudan. In addition, the predominant manifestations of severe malaria were hypotension (27.3%), cerebral malaria (16.5%), and convulsions (13.0%), and their frequency was not significantly different between patients with *P. falciparum* or *P. vivax* malaria. It has been demonstrated that severe malarial anaemia was the most common complication (45.4%) of *P. falciparum* malarial infection among patients in a hospital (Gedarif) in eastern Sudan [8]. The difference in dominant complications observed between the two studies may be attributable to the age difference, which ranged between two and four years in the Gedarif study compared with a range of 17–78 years, in the current study [8]. Anaemia was the most common manifestation (30%), followed by hypoglycaemia, of severe malaria among pregnant women in the same study area [6]. Both clinical manifestations (anaemia and hypoglycaemia) were less prominent in the current study, perhaps because pregnant patients were more vulnerable to anaemia and hypoglycaemia than adult non-pregnant patients.

Interestingly, it has been observed that 36.2% of adult patients in the same study area had anaemia regardless of their age, sex, education level or infection with malaria [12]. In Tanzania, the clinical manifestations of severe malaria in children younger than five years were cerebral malaria (47.3%) and severe anaemia (14.6%) [4]. Severe anaemia (8.57%) and circulatory collapse (11.90%) were the most common manifestations of severe malaria in neighbouring Ethiopia [13]. In Southeast Asia, which is characterized by unstable malaria transmission, the incidence of anaemia and convulsions decreased with age, whereas the incidence of hyperparasitaemia, jaundice, and renal insufficiency increased with age [14]. In India, acute renal failure and jaundice were more common in adults whereas children frequently developed severe anaemia, while cerebral malaria occurred equally in adults and children [15]. One tenth (10.8%) out of these 139 patients presented with hypoglycaemia. It was previously shown that 20% and 16% of children with severe malaria in Malawi and Kenya, respectively, had hypoglycaemia before treatment [16,17]. Hypoglycaemia is one of the defining features of severe malaria according to WHO guidelines, and indicates a poor prognosis [2]. Furthermore, it is a treatable cause of other features of severe malaria e g, coma and convulsions, especially in severe childhood illnesses [2]. Recent findings showed a significant difference between blood glucose levels between children who died and survivors [18].

The manifestations of severe malaria were not significantly different between *P. falciparum* and *P. vivax* malaria in this report. Previously, *P. vivax* was considered the cause of tertian benign malaria that rarely led to a severe form of the disease. However, increasing evidence has shown an increased risk of mortality and morbidity owing to *P. vivax* malaria [10,19-22]. Generally, most reports on severe *P. vivax* malaria are from Southeast Asia and India, and there have been few published data on severe *P. vivax* from Africa. It has been documented different manifestations of severe *P. vivax* malaria among children in a nearby hospital (New Halfa) [10]. The clinical presentation of severe malaria caused by *P. vivax* in India presented with cerebral malaria, severe anaemia, renal failure, hypoglycaemia, jaundice, acute respiratory distress syndrome, shock, and death [20]. However, to the best of the knowledge, there have been no reports from African countries regarding adult patients with malaria. Of note, there are no specific manifestations or treatment for severe *P. vivax* malaria, but according to the WHO guidelines, it should be considered as severe as *P. falciparum* malaria [11].

In the current study, three patients (2.0%) died owing to severe *P. falciparum* malaria. In Tanzania, the fatality rate of severe malaria was 3.2% and the majority of deaths occurred in children younger than five years [4]. High mortality rates (33.3%), mainly in young adults, were observed among patients with severe malaria at Dakar, Senegal [23]. Likewise, a high fatality rate (24.1%) was observed among 1,050 patients with severe malaria in Southeast Asia, where mortality increased from 6.1% in children to 36.5% in patients aged >50 years [15]. Low death rates may be caused by underestimation, as some cases of severe malaria might have died at home without access to hospital treatment. Furthermore, the low rate of hypoglycaemia in this study could explain the low mortality rate itself where recent study showed a significant difference between blood glucose levels between children who died and survivors [18]. It is also possible that genetic factors might explain the mortality in this setting.

There are some limitations of this study; manifestations of the disease itself were overlaps and might not be strictly followed e.g. hypotension is only severe disease if not responsive to fluids, bleeding by itself is not severity as well, unless it is followed by circulatory collapse and hyperbilirubinaemia is considered a severity criterion if its followed by other organ dysfunctions. Furthermore PCR was not performed in any sample of the patients to confirm the parasite species as has been done in some other setting [24].

## Conclusions

Severe malaria due to *P. falciparum* or *P. vivax* malaria is an entity that exists among adults in eastern Sudan, with similar manifestations between severe *P. falciparum* and *P. vivax*.

## Competing interests

The authors declare that they have no competing interests.

## Authors’ contributions

TMA and IA designed the study and shared in the statistical analyses. MTA and ISA conducted the clinical work. HZH and MMM conducted the biochemical work. All the authors shared in the draft and approved the manuscript.
